# *In vitro* antiplasmodial and cytotoxic properties of some medicinal plants from western Burkina Faso

**DOI:** 10.4102/ajlm.v2i1.81

**Published:** 2013-03-08

**Authors:** Souleymane Sanon, Adama Gansane, Lamoussa P. Ouattara, Abdoulaye Traore, Issa N. Ouedraogo, Alfred Tiono, Donatella Taramelli, Nicoletta Basilico, Sodiomon B. Sirima

**Affiliations:** 1 Centre National de Recherche et de Formation sur le Paludisme, Ouagadougou, Burkina Faso; 2 Dipartimento di Scienze Farmacologiche e Biomolecolari (DiSFeB), Università di Milano, Italy; 3 Dipartimento di Scienze Biomediche, Chirurgiche e Odontoiatriche, Università di Milano, Italy

## Abstract

**Background:**

Resistance of malaria parasites to existing drugs complicates treatment, but an antimalarial vaccine that could protect against this disease is not yet available. It is therefore necessary to find new effective and affordable medicines. Medicinal plants could be a potential source of antimalarial agents. Some medicinal plants from Burkina Faso were evaluated for their antiplasmodial and cytotoxic properties *in vitro*.

**Methods:**

Crude dichloromethane, methanol, water-methanol, aqueous and alkaloids extracts were prepared for 12 parts of 10 plants. Chloroquine-resistant malaria strain K1 was used for the *in vitro* sensibility assay. The *Plasmodium* lactacte dehydrogenase technique was used to determine the 50% inhibitory concentration of parasites activity (IC_50_). The cytotoxic effects were determined with HepG2 cells, using the tetrazolium-based colorimetric technique, and the selectivity index (SI) was calculated.

**Results:**

Sixty crude extracts were prepared. Seven extracts from *Terminalia avicenoides* showed IC_50_ < 5 µg/mL. The IC_50_ of dichloromethane, methanol, aqueous and alkaloids extracts ranged between 1.6 µg/mL and 4.5 µg/mL. Three crude extracts from *Combretum collinum* and three from *Ficus capraefolia* had an IC_50_ ranging between 0.2 µg/mL and 2.5 µg/mL. Crude extracts from these three plants had no cytotoxic effect, with SI > 1. The other plants have mostly moderate or no antimalarial effects. Some extracts from *Cordia myxa*, *Ficus capraefolia* and *Opilia celtidifolia* showed cytotoxicity, with an SI ranging between 0.4 and 0.9.

**Conclusion:**

Our study showed a good antiplasmodial *in vitro* activity of *Terminalia avicenoides, Combretum collinum* and *Ficus capraefolia*. These three plants may contain antiplasmodial molecules that could be isolated by bio-guided phytochemical studies.

## Introduction

Malaria remains a serious worldwide health problem due to the emergence and spread of parasite resistance to well-established antimalarial drugs and mosquito vectors resistant to insecticides.^1^ In sub-Saharan African populations, malaria is one of the diseases causing most morbidity and mortality. It is estimated that each year malaria causes nearly 800 000 deaths,^2,3^ mostly of African children aged below 5 years. In Burkina Faso, malaria is the leading cause of hospitalisation, with 2 million cases per year, and it remains also the principal cause of death in local health centers, with a rate of 50.7% in 2009.^4^ Although continued attempts to develop a vaccine for malaria are ongoing, distribution of mosquito nets, household spraying, and prophylaxis remain the primary prevention methods and antimalarial drugs remain the only treatment option.^5^ In order to decrease the risk of chemo-resistance to most of the antimalarial drugs, the World Health Organization (WHO) has recommended artemisinin-based combination therapies (ACTs) for the management of uncomplicated *P. falciparum* malaria cases. Unfortunately, ACT treatment failures have been reported in some countries.^6,7,8,9^ In addition, these drugs are expensive, limiting their use in a population with average annual income around $100. Thus the use of traditional and less expensive preparations is common.^10^ Historically, many drugs effective against parasitic diseases stem from traditional medicine, such as quinine and artemisinin.^11,12,13^ Today, 30% of drugs on the pharmaceutical market come from nature^14^ and medicinal plants constitute a popular source of potential antimalarial agents. In the in western area of Burkina Faso, ten medicinal plants are widely used by traditional healers,^15^ although little scientific data exist on their effectiveness in treating malaria. The aim of this study is to determine which of these plants show promising antimalarial effects as well as low toxicity *in vitro*, paving the way for potential pharmaceutical development.

## Material and methods

This study was approved by the national ethical committee to be conducted with the traditional healers on the study site.

### Study site and plants

The study was conducted in the province of Comoe, where malaria transmission is continuous and which covers 15 871 km^2^ with 277 384 inhabitants. With an average annual rainfall of 900 mm, this area is one of the wetter regions of the country, and has a large plant biodiversity. The herbal pharmacopoeia has expanded rapidly in this province, and traditional healers collaborate with researchers in the field of herbal medicine. Twelve samples consisting of leaves and bark from ten plants (*Terminalia avicenoides, Combretum collinum, Ficus capraefolia, Anthocleista nobilis, Celtis integrifolia, Cordia myxa, Lophira lanceolata, Oppilia celtidifolia, Securinega virosa* and *Tapinanthus dodoneifolius*) were identified. The scientific identification and the herbarium were made by botanical taxonomists from the Centre National de Recherche Scientifique *et* Technologique (CNRST) of Burkina Faso. The specimen voucher number from this herbarium was deposited in Centre National de Recherche et de Formation sur le Paludisme (CNRFP). These parts of plants were used by traditional healers to treat malaria-like symptoms. Samples were harvested during the rainy season, washed, dried and pulverised for phytochemical extractions.

### Crude extracts preparation

Extraction based on maceration, decoction and preferential depletion methods were used to obtain five types of extract for each of the 12 plant parts. We obtained three organic extracts, one aqueous with water, and one crude alkaloid. Crude organic extracts were prepared by maceration for 16 hours successively with dichloromethane (CH_2_Cl_2_), methanol (CH_3_OH) and water-methanol (CH_3_OH/H_2_O) solvents. Plant powder (20 g) was used for these organic extraction methods with 500 mL of each solvent. CH_2_Cl_2_ extract was air dried at room temperature. CH_3_OH and CH_3_OH/H_2_O extracts were freeze-dried with lyophilisator (Brand) after total evaporation of solvents. Aqueous extracts were prepared by boiling 10 g of plant powder in 500 mL of purified water for 30 minutes. After cooling, solutions were filtered on cotton wool and freeze-dried. Crude alkaloid extracts were obtained by alkalinisation with NH_4_OH of the plant powder and extraction with CH_2_Cl_2_ for 24 hours. Plant powder (20 g) was used by applying the classical alkaloids extraction method.^16^ After 16 hours of maceration with ammoniac and CH_2_Cl_2_, a percolation was made with CH_2_Cl_2_ solvent. Then 500 mL of dichloromethane layer was concentrated under vacuum and then extracted with a 2% solution of H_2_SO_4_. The aqueous acid solution was alkalinised again with NH_4_OH and extracted with CH_2_Cl_2_, and a crude alkaloids extract was obtained by concentration.

Each of the 60 extracts was dissolved in appropriate solvent of dimethyl sulfoxide (DMSO) and purified water (DMSO/water) to give a homogeneous solution at an initial concentration of 1 mg/mL. Samples were then serially diluted with complete culture media (RPMI 1640 with albumax) to achieve the required concentration with DMSO concentration < 0.5%.

### *In vitro* antiplasmodial assays

#### *Plasmodium falciparum* culture

Sixty extracts were tested for their effectiveness in preventing growth of the most common chloroquine-resistant strain of malaria found in Burkina Faso, *Plasmodium falciparum* K1. The malaria strains were provided by the London School of Tropical Medicine and Hygiene (England) and were grown under standard conditions as previously described.^17^ They were maintained in continuous culture at the CNRFP, in a sterile atmosphere under a laminar flow hood in 200 µL blood group O+ using RPMI 1640 (MegaCell, Sigma Aldrich, USA) medium (5 mL) supplemented with 2-hydroxyethlpiperazine-N-2ethanesulfonic acid (HEPES acid) (25 mM; Gibco-BRL, Paisley, Scotland), NaHCO_3_ (25 mM), 1% Albumax and washed erythrocytes to yield a final haematocrit of 4%. Parasitaemia was maintained between 1% and 6% by dilution with non-infected O+ erythrocytes. Blood was obtained from subjects who had not received antimalarial treatment during the previous two weeks and had an AA electrophoresis (no sickle cell trait). The 75 cm^3^ culture flasks were incubated for 24 hours at 37 °C in a CO_2_ incubator (HeraCell 150, Forma Scientific), with 2% O_2_, 5% CO_2_, 93% N_2_ and 90% humidity. Every 24 hours, supplemented media was renewed and parasitemia was checked by blood smear with optical microscope.

#### Evaluation of antiplasmodial effect of extracts

Antimalarial effects were quantified with respect to inhibition of parasite growth, as measured by the production of *Plasmodium* lactate dehydrogenase (pLDH). Testing was performed in three steps in duplicate in 96-well flat bottom plates (TPP, Switzerland).

Malaria parasites were cultured with seven different concentrations of each extract and parasite growth was assessed by the production of pLDH. Each extract was applied in a series of seven duplicate dilutions (final concentrations ranging from 0.78 µg/mL to 50 µg/mL) on two rows. 100 µL of asynchronous parasitised erythrocytes at a hematocrit of 2% with parasitemia between 1.5% and 2% were prepared in 100 µL of each extract. The plates were then placed at 37 °C in a modular incubator chamber with a humidified atmosphere of the same gas mixture as above for 72 hours. Dihydroartemisinin was used to validate the malaria test and chloroquine diphosphate salt (Sigma Aldrich) was used to validate the real chloroquine resistance of malaria strain K1. Infected and uninfected erythrocytes O+ were used as positive and negative controls, respectively. Parasite growth was determined by measuring the content of parasite lactate dehydrogenase^18^ using Malstat, NTB/PES reagents. The microplates were read with a spectrophotometer (Biotek EL x 808) at a wavelength of 650 nm. Absorbance data were entered into Microsoft Excel to calculate the percent inhibition relative to positive control from the mean of raw data for each concentration. Table Curve version 5.0 software was used to plot inhibition curves and calculate the inhibition concentration of drug that reduced the pLDH activity by 50% (IC_50_). The results were categorised following Deharo’s et al classification:^19^ good antimalarial effect IC_50_ < 5 µg/mL, moderate antimalarial effect 5 µg/mL ≤ IC_50_ < 10 µg/mL and inactive extract IC_50_ ≥ 10 µg/mL.

### *In vitro* cytotoxicity assay of extracts on human cells

Human hepatoma cells ATCC # HB-8065 (HepG2) were obtained from the American Type Culture Collection (ATCC, Manassas, USA). The cells were grown in RPMI-1640 (MegaCell, Sigma Aldrich, USA) supplemented with 10% Fetal Bovine Serum (v/v), 1% mix (v/v) of 200 mM L-glutamine, 10 000 IU/mL penicillin and 10 mg/mL streptomycine, (GibcoBRL) within a humidified atmosphere of 6% CO_2_ and 94% de N_2_ at 37 °C over 5 days. Cultures were maintained by sub-culturing flasks every 4 days at 5 × 10^4^ cells/25 cm^2^ flask by trypsination. The cytotoxicity of the extracts was assessed using a tetrazolium salt MTT (3-[4.5-dimethylthiazol-2-yl]-2.5-diphenyltetrazolium bromide) (Sigma) colorimetric method, based on reagent cleavage by mitochondrial dehydrogenase in viable cells.^20^ Growing cells were placed at a density of 5 × 10^4^ cells per well in microplates in 100 µL of culture medium. The microplates were incubated in a humidified atmosphere of 6% CO_2_ and 94% N_2_ at 37 °C for 24 hours. For each extract, seven duplicate dilutions were prepared with concentrations ranging from 0.78 µg/mL to 50 µg/mL in DMSO and then in medium culture. For each test, we used a growth (cells plus medium only), positive (with doxorubicin) and a negative control (with DMSO) respectively. Cytotoxicity was scored as the percentage reduction in absorbance at 570 nm versus that of the untreated control culture. A selectivity index (SI), corresponding to the ratio between antimalarial and cytotoxic properties, was calculated according to the following formula: SI_extract_ = IC_50cytotoxic_/IC_50malaria_. The extract was considered to have negligible cytotoxicity if SI > 1.^21^

## Results

### Antimalarial effects

Among the 60 extracts tested, 21 were identified as having good antimalarial effects (IC_50_ < 5 µg/mL), 14 with moderate effects (5 µg/mL≤ IC_50_ < 10 µg/mL), and 25 as inactive (IC_50_ ≥ 10 µg/mL) (Table 1). *Terminalia avicenoides* produced the most effective antimalarial extracts, with four coming from its leaves and three from its stem bark (Table 2). Three extracts from *Combretum collinum* and three from *Ficus capraefolia* also had good antimalarial effects (Table 2). Extracts from the seven other plants had mostly moderate to little effect.

**TABLE 1:** *In vitro* antimalarial and cytotoxic effects of indigenous plant extracts.

Plant Species (Botanical family)	Local name	Administration method	Plant part	Extract	IC_50_ (K1) (µg/mL)	Level of antimalarial effect^a^	SI	Level of cytotoxic risk^b^
*Terminalia avicennioides* (Combretaceae)	Kù’nhil- blï’ngù (G), Kokogo (K)	Oral, Body bath	Leaves	CH_2_Cl_2_	1.6	Good	32.0	Low
				CH_3_OH	1.9	Good	26.2	Low
				CH_3_OH/H_2_O	5.4	Moderate	9.2	Low
				H_2_O	2.6	Good	19.4	Low
				alkaloids	1.2	Good	43.1	Low
			Bark	CH_2_Cl_2_	3.6	Good	13.7	Low
				CH_3_OH	4.5	Good	11.0	Low
				CH_3_OH/H_2_O	7.4	Moderate	6.8	Low
				H_2_O	6.8	Moderate	7.3	Low
				alkaloids	2.9	Good	17.3	Low
*Combretum collinum* (Combretaceae)	Kagan-ga (M)	Oral, Body bath	Leaves	CH_2_Cl_2_	0.2	Good	140.2	Low
				CH_3_OH	11.2	Inactive	3.2	Low
				CH_3_OH/H_2_O	2.1	Good	21.1	Low
				H_2_O	38.4	Inactive	1.3	Low
				alkaloids	0.4	Good	113.6	Low
*Ficus capraefolia* (Moraceae)	Ka funa sô (D)	Oral, Body bath, Steam bath	Leaves	CH_2_Cl_2_	1.8	Good	27.0	Low
				CH_3_OH	2.5	Good	4.9	Low
				CH_3_OH/H_2_O	12.5	Inactive	2.0	Low
				H_2_O	13.1	Inactive	0.4	High
				alkaloids	0.9	Good	52.6	Low
*Anthocleista nobilis* (Loganiaceae)	Falatô-dêbê (D), Djântongù (T)	Oral, Body bath, Steam bath	Leaves	CH_2_Cl_2_	10.0	Moderate	5.0	Low
				CH_3_OH	30.2	Inactive	1.6	Low
				CH_3_OH/H_2_O	20.7	Inactive	1.2	Low
				H_2_O	38.4	Inactive	1.3	Low
				alkaloids	1.8	Good	27.0	Low
*Celtis integrifolia* (Ulmaceae)	Kanga (M)	Oral, Body bath	Leaves	CH_2_Cl_2_	3.7	Good	13.4	Low
				CH_3_OH	46.8	Inactive	1.1	Low
				CH_3_OH/H_2_O	47.5	Inactive	0.5	High
				H_2_O	20.6	Inactive	2.4	Low
				alkaloids	10.8	Inactive	4.6	Low
*Cordia myxa* (Boraginaceae)	Dàmàtéré (T)	Oral, Body bath, Steam bath	Leaves	CH_2_Cl_2_	6.2	Moderate	8.0	Low
				CH_3_OH	21.6	Inactive	0.9	High
				CH_3_OH/H_2_O	94.6	Inactive	0.5	High
				H_2_O	15.4	Inactive	3.2	Low
				alkaloids	4.2	Good	11.8	Low
*Lophira lanceolata* (Ochnaceae)	Nô’ng-plà’ng (K)	Oral, Body bath	Leaves	CH_2_Cl_2_	4.7	Good	10.5	Low
				CH_3_OH	38.4	Inactive	1.3	Low
				CH_3_OH/H_2_O	22.1	Inactive	2.3	Low
				H_2_O	12.5	Inactive	3.9	Low
				alkaloids	5.9	Moderate	8.5	Low
			Bark	CH_2_Cl_2_	5.5	Moderate	9.1	Low
				CH_3_OH	9.8	Moderate	9.5	Low
				CH_3_OH/H_2_O	14.7	Inactive	1.7	Low
				H_2_O	4.7	Good	5.3	Low
				alkaloids	2.5	Good	19.8	Low
*Opilia celtidifolia* (Opiliaceae)	Ku’nhil-blingù (G)	Oral, Body bath	Leaves	CH_2_Cl_2_	2.8	Good	17.4	Low
				CH_3_OH	16.2	Inactive	3.1	Low
				CH_3_OH/H_2_O	61.2	Inactive	0.4	High
				H_2_O	15.1	Inactive	3.3	Low
				alkaloids	6.9	Moderate	7.2	Low
*Securinega virosa* (Euphorbiaceae)	Sï’ngnamâ (G)	Oral, Body bath	Leaves	CH_2_Cl_2_	7.1	Moderate	7.0	Low
				CH_3_OH	7.6	Moderate	2.7	Low
				CH_3_OH/H_2_O	9.7	Moderate	1.8	Low
				H_2_O	14.5	Inactive	3.5	Low
				alkaloids	1.6	Good	31.8	Low
*Tapinanthus dodoneifolius* (Loranthaceae)	Sï-làdon (D)	Oral, Body bath, Steam bath	Leaves	CH_2_Cl_2_	6.5	Moderate	7.7	Low
				CH_3_OH	5.2	Moderate	7.4	Low
				CH_3_OH/H_2_O	20.6	Inactive	1.2	Low
				H_2_O	43.7	Inactive	1.1	Low
				alkaloids	11.3	Inactive	4.4	Low

SI, selectivity index; D, Dioula; G, Goin; T, Turka; K, Karaboro; M, Mossi; B, Bobo.

Level of antimalarial effect^a^: good, IC_50_ < 5 ug/mL; moderate, 5 ug/mL ≤ IC_50_ < 10 ug/mL; inactive, IC_50_ ≥ 10 ug/mL.

Level of cytotoxic risk^b^: low, SI > 1; high, SI < 1.

**TABLE 2:** Promising crude extracts from medicinal plants according to antiplasmodial activity level and cytotoxicity.

Plant species	Antiplasmodial activity level			Cytotoxicity (IS > 1)
	Good (IC_50_ < 5 µg/mL)	Moderate (5 ≤ IC_50_ < 10 µg/mL)	Low (IC_50_ ≥ 10 µg/mL)	
*Terminalia avicenoides*	Ext. DC (leaves, bark) Ext. MT (leaves, bark) Ext. AQ (leaves) Ext. ACE (leaves, bark)	Ext. HMT (leaves, bark) Ext. AQ (bark)	No extracts	No extract (7.3 ≤ IS ≤ 43.1)
*Combretum collinum*	Ext. DC (leaves) Ext. DMT (leaves) Ext. ACE (leaves)	No extracts	Ext. MT (leaves) Ext. AQ (leaves)	No extract (1.3 ≤ IS ≤ 140.2)
*Ficus capraefolia*	Ext. DC (leaves) Ext. MT (leaves) Ext. ACE (leaves)	No extracts	Ext. DMT (leaves) Ext. AQ (leaves)	No extract (2.0 ≤ IS ≤ 52.6)

Ext. DC, Dichloromethane extract; Ext. MT, Methanol extract; Ext. HMT, Hydro methanol extract, Ext. AQ, Aqueous extract; Ext. ACE, Crude alkaloids extract.

### Toxicity

The extracts with antiplasmodial effects from *Terminalia avicenoides, Combretum collinum* and *Ficus capraefolia*, had low risk of cytotoxicity (SI > 1), with SI ranging from 4 to 140 (Table 1). Higher risk of cytotoxicity (SI < 1) was found with five extracts from four plants: a water-methanol extract from *Celtis integrifolia* (SI > 0.5), methanol and water-methanol extracts from *Cordial myxa* (SI = 0.9 and SI = 0.5, respectively), aqueous extract from *Ficus capraefolia* (SI = 0.4) and water-methanol extract from *Opilia celtidifolia* (SI = 0.4).

## Discussion

The best antimalarial effects were obtained with extracts of three plants, namely, *Terminalia avicenoides, Combretum collinum* and *Ficus capraefolia*. The four crude extracts from the leaves and three from the stem bark of *Terminalia avicenoides* showed good antimalarial effects against the CQ-resistant strain K1, with IC_50_ values ranging between 1.16 µg/mL to 4.53 µg/mL. In Nigeria, a similar study showed that methanol extracts of leaves of *Terminalia avicennoides* had an IC_50_ = 14.09 µg/mL with the K1 malaria strain.^22^

Based on Deharo’s efficiency criteria, results from Nigeria are different from our findings. These differences may be related to many parameters, including the local environment and the collection periods, which contribute to the variation of plant chemical components as shown in a previous study on seasonal effects on bioactive compounds.^23^ The biological results could be linked to the laboratory techniques used. In phytochemical studies, extraction methods are often different because the ability of extracting a solvent of chemical groups is related to its polarity. Concerning biological assessment, the results are often based on the sensitivity of techniques used. A previous study done in Australia pointed to the difference of sensitivity of the Flow cytometry assay, the Sybr Green plate reader assay, the *Plasmodium falciparum* lactate dehydrogenase assay and light microscopy used in *in vitro* antiplasmodial activity assessments.^24^

Despite the difference of IC_50_ values obtained, which could be related to laboratory sensitivity techniques and environmental conditions, the antiplasmodial properties of *Terminalia avicenoides* were promising. Our study confirms the pharmacological properties of this plant species shown by its antifungal activity against *Candida albicans*^25^ and parasitogical activity on *Trypanosoma mali*.^26^ Another study showed good antidiarrheal properties for an aqueous extract of the roots of *Terminalia avicennoides*.^27^ It is recommended that future studies should consider this plant as a potential source of antiplasmodial molecules. Our study appears to be the first to demonstrate the antimalarial effects of this plant.

With *Combretum collinum*, dichloromethane, water-methanol and alkaloid extracts of leaves had good antimalarial activity, with IC_50_ values ranging from 0.2 µg/mL to 2.14 µg/mL. It appears that our findings are the first report of extracts exhibiting *in vitro* antiplasmodial activities. However, studies have shown the pharmacological property of some species of the same botanical family (Combretaceae). Acetone extracts from *Combretum molle*, which belongs to the same botanical family, have shown good antimalarial effects, with IC_50_ values from 2.2 µg/mL^28^ to 8.2 µg/mL^28^ and selective inhibition effect of HIV-1 replication.^29^ The plant also has antifungal, anti-inflammatory and antilarvicidal properties.^30,31,32^ According to these results, the antimalarial effect obtained with *Combretum collinum* can be explained by the presence of chemical groups specific to this botanical family.

*Ficus capraefolia* is the third plant which has good antimalarial effect, with dichloromethane, methanol and crude alkaloid extracts (IC_50_ < 5 µg/mL ranging between 0.95 µg/mL and 2.85 µg/mL). Our study appears to be the first report to show the antimalarial activity of this plant. Other studies have shown pharmacological properties of other species of the same botanic family (Moraceae).

Thus, *Ficus sycomorus* (L) had a moderate antimalarial effect with IC_50_ < 10 µg/mL,^16^ and *Ficus sur* (Forssk) was inactive, with IC_50_ = 27.4 µg/mL and IC_50_ > 100 µg/mL using chloroquine resistant strains ENT 30 and VI/S respectively.^33^ In the other species of Moraceae, which is *Ficus fistulosa* Reinw, molecules such as the flavonoid artonin F, flavonoid 7-*demethylartonol* and flavonoid cycloartobiloxanthone have been isolated.^34,35^ The pharmacological effects that our study and others show could be attributed to this chemical group, but a bioguided phytochemical study would be necessary to verify whether it has the same antimalarial molecules as the ones found in our species.

Besides the three previous plant species, *Anthocleista nobilis*, *Opilia celtidifolia*, *Lophira lanceolata*, *Securinega virosa* and *Tapinanthus dodoneifolius* all showed moderate antimalarial activity. A literature search on the antimalarial activities of the aforementioned plants has shown no data. These pharmacological properties could be attributed to alkaloids which could be contained in this plant.^36,37^ Analgesic properties^38^ and antibacterial activity^39^ of ethanol and methanol extracts have been shown in *Cordia mixa*.

In Kenya, aqueous and methanol extracts from leaves of *Securinega virosa* showed antimalarial activity using the CQ-resistant strain D6 with IC_50_ of 25.52 µg/mL and 2.28 µg/mL respectively.^40^ Our study confirms the antimalarial property shown by the Kenyan study, using another CQ-resistant strain K1. Otherwise, this antimalarial effect could be attributed to virosecurinine, an alkaloid isolated from its leaves.^41^ Our study appears to be the first report showing a moderate antimalarial effect for *Lophira lanceolata* but its antimicrobial activity against *Candida albicans* has been described previously in Cameroun.^42^ In Nigeria, a study showed that a methanol extract of the leaves of *Tapinanthus dodoneifolius* has very good antimicrobial properties.^43^

The promising antimalarial extracts from *Terminalia avicenoides, Combretum collinum* and *Ficus capraefolia* showed no cytotoxic effect, with a high selectivity index > 10. This finding is in concordance with some cytotoxicity studies of extracts from African medicinal plants.^44,45^ Otherwise, among the extracts which have moderate and no antimalarial effects, the risk of cytotoxicity for human cells has been reported, as shown by water-methanol extracts from *Celtis integrifolia* (SI > 0.5), methanol and water-methanol extracts from *Cordia myxa* (respectively SI = 0.9 and SI = 0.5), aqueous extracts from *Ficus capraefolia* (SI = 0.4) and water-methanol extracts from *Opilia celtidifolia* (SI = 0.4).

This shows that the toxicity of a drug depends on effective dose used. Furthermore, it is also important that even though they are effective in treating malaria, the cytotoxicity of to these plants must be taken into account especially by traditional healers for whom the major issue is giving the correct dosage. As shown in previous studies, in traditional medicine practice, medicinal plants can be used but the dosage must be taken into consideration.^46,47^ The results of this study can improve the traditional use of plants and protect people from risks following their administration.

It appears for the first time from scientific investigation that *Terminalia avicennoides, Ficus capraefolia* and *Combretum collinum* may be potential sources of antimalarial agents due to their good antimalarial effects and the lack of cytotoxic effects of their extracts. The seven other plants have mostly moderate antimalarial effects but some extracts from *Celtis integrifolia*, *Cordial mixa* and *Opilia celtidifolia* showed cytotoxic effects. These plants can be used in traditional medicine by paying close attention to the dosage. The promising results obtained can be a starting point to seek bioactive compounds by bioguided fractionation and biological studies, for the development of new drugs.
